# Ultrasound Elastography for the Assessment of Sarcopenia

**DOI:** 10.3390/jcm15072566

**Published:** 2026-03-27

**Authors:** Chenzi Zhang, Lin Kang

**Affiliations:** Department of Geriatrics, Peking Union Medical College Hospital, Chinese Academy of Medical Sciences & Peking Union Medical College, Beijing 100730, China; qhzhangjia@163.com

**Keywords:** sarcopenia, shear-wave elastography, muscle quality, assessment

## Abstract

**Background**: Sarcopenia is an age-related syndrome characterized by progressive loss of skeletal muscle mass and strength, representing a major contributor to disability and increased mortality in older adults. Current diagnostic frameworks increasingly emphasize muscle quality alongside quantity, creating a clinical need for bedside tools that can objectively assess muscle mechanical properties. Shear-wave elastography (SWE), an ultrasound-based technique that quantifies muscle stiffness, has emerged as a promising biomechanical biomarker of muscle quality. **Aim**: This narrative review evaluates the evidence supporting SWE for assessing muscle quality and its association with aging, sarcopenia, and functional outcomes. **Methods**: We searched PubMed, Embase, and Web of Science (from January 2010 to December 2025) using terms related to elastography and sarcopenia. Based on relevance and methodological quality, approximately 50 key studies were selected for in-depth discussion and synthesis. **Synthesis**: Observational studies consistently demonstrate that SWE detects age-related reductions in muscle stiffness, which correlate significantly with declines in muscle strength and physical performance. Unlike conventional B-mode ultrasound, which primarily provides morphological parameters, SWE directly reflects intrinsic tissue mechanics, enabling more direct assessment of muscle quality. In high-risk populations such as patients with type 2 diabetes, reduced muscle stiffness is also associated with sarcopenia and poor functional outcomes. However, reported stiffness trends with aging remain heterogeneous, and validated diagnostic thresholds are lacking. Stiffness changes vary by muscle group, acquisition protocol, and loading state. Clinical implementation is currently limited by inter-device variability, operator dependence, and sensitivity to muscle loading conditions. **Conclusions**: Current evidence suggests that SWE holds promise as an adjunctive research tool for assessing muscle quality and risk stratification, but it is not yet ready for standalone clinical diagnosis due to methodological heterogeneity, lack of validated cutoffs, and limited longitudinal data. Future large-scale, longitudinal, multicenter studies with standardized protocols are needed to establish its definitive diagnostic utility.

## 1. Introduction

Sarcopenia is defined as an age-related, generalized, and potentially reversible disease characterized by loss of skeletal muscle mass and strength [1]. In contrast, dynapenia refers specifically to age-associated loss of muscle strength not caused by neurologic or muscular diseases. Dynapenia predisposes older adults to increased risk of functional limitations and mortality [2].

Recent international consensus statements, including the Asian Working Group for Sarcopenia (AWGS) 2025 update, have refined its diagnostic framework, which is now centered on the concurrent presence of low muscle mass and low muscle strength, while measures of physical performance are categorized as outcome indicators rather than diagnostic components [3]. This conceptual shift highlights the central role of muscle quality in explaining age-related decline in strength that disproportionately exceeds loss of muscle mass. Muscle quality is increasingly recognized as a multidimensional health indicator, encompassing morphological features (e.g., intramuscular adipose infiltration, tissue density, architectural integrity) and functional properties (e.g., metabolic efficiency, regenerative capacity, mitochondrial function, and protein turnover). As articulated in the AWGS 2025 “Muscle Health Framework,” muscle functions as an energy reservoir and endocrine organ, reinforcing the systemic importance of these functional attributes [3]. This integrated view is supported by recent Delphi survey findings, which identified muscle mass, muscle strength, and muscle-specific strength (force generated per unit mass) as core components of sarcopenia [1]. Within this framework, muscle quality serves as the foundational determinant of muscle strength, power, and overall physical function, bridging the gap between tissue morphology and physiological performance.

At the molecular level, microRNAs (miRNAs) have been identified as key regulators of myogenesis and inflammation in sarcopenia [4]. Emerging evidence further demonstrates that specific miRNA signatures—such as dysregulation of myomiRs (miR-1, miR-133, miR-206) and inflammation-related miRNAs—are closely associated with muscle quality deterioration and phenotypes like sarcopenic obesity [5]. These circulating or tissue-based miRNA alterations reflect underlying pathological processes including myosteatosis and inflammatory activation, positioning miRNAs as promising early biomarkers for muscle quality decline [5]. Therefore, a comprehensive assessment of muscle health should integrate multi-level information, from macroscopic imaging features to these underlying molecular signatures.

Established methods for assessing muscle quality include computed tomography (CT), dual-energy X-ray absorptiometry (DXA), and magnetic resonance imaging (MRI). CT is often regarded as a reference standard and has been used to establish population-specific cutoffs, such as age- and sex-stratified psoas muscle index values at the L3 vertebra in healthy Asian adults [6]. In clinical practice, DXA is widely adopted and endorsed by consensus guidelines including AWGS 2025, which provides validated diagnostic thresholds (e.g., appendicular skeletal muscle index < 7.0 kg/m^2^ for men and <5.4 kg/m^2^ for women [3]. Although MRI is recognized as an advanced modality capable of assessing both muscle quantity and quality [7], its clinical utility in sarcopenia is limited by cost, accessibility, and lack of standardized diagnostic protocols.

At the bedside, conventional B-mode ultrasonography offers a more accessible alternative for evaluating muscle morphology. However, similar to the aforementioned modalities, its standard parameters (e.g., thickness, cross-sectional area) are fundamentally limited to assessing muscle quantity and provide only indirect correlation with function. They fail to capture muscle quality—a key determinant of strength and metabolic health governed by the tissue’s intrinsic biomechanical properties. Therefore, advancing sarcopenia diagnostics requires a paradigm shift toward techniques that can objectively quantify these intrinsic mechanical properties, moving beyond anatomical measurements alone. This shift reflects the fundamental distinction where traditional ultrasound imaging primarily focuses on providing anatomical details, whereas shear-wave elastography emerges as a functional technique for biomechanical assessment [8]. Ultrasound elastography addresses this need. The clinical adoption and maturity of shear-wave elastography (SWE) vary substantially across different organs. In chronic liver diseases, especially non-alcoholic fatty liver disease, it serves as a core non-invasive tool for managing chronic liver diseases [9,10,11]. In the thyroid, SWE acts as a supplement to conventional ultrasound [12]. Moreover, it has potential to assess renal stiffness in chronic kidney diseases [13].

These applications have established elastography as a robust, quantitative imaging technique capable of reflecting underlying tissue composition and microstructural remodeling. However, its application to skeletal muscle—despite being a highly dynamic and mechanically active tissue—has only recently become a focus of elastography research, particularly in the context of aging and sarcopenia. This review aims to synthesize the current evidence on SWE for assessing muscle quality and its association with functional outcomes in aging and sarcopenia.

## 2. Literature Search

This narrative review summarizes the current status of ultrasound elastography in skeletal muscle aging and its potential value in sarcopenia diagnosis. We searched PubMed, Embase, and Web of Science for articles published from January 2010 to December 2025, using combinations of terms related to the technique (“elastography”, “shear-wave elastography”, “SWE”) and the condition (“sarcopenia”, “muscle quality”, “muscle stiffness”, “aging”). Reference lists of relevant reviews were manually screened. After removing duplicates, titles and abstracts were screened, yielding over 180 full-text articles for review. Studies were selected based on representativeness, methodological quality, and direct relevance to the research questions. Priority was given to original research and high-quality reviews that provided quantitative insights into SWE-derived metrics and their associations with muscle strength, physical performance, or sarcopenia. While the primary focus was skeletal muscle SWE, selected studies on other tissues (e.g., liver, diaphragm) and elastography techniques (e.g., strain elastography) were included for contextual background.

From the over 180 full-text articles reviewed, approximately 50 studies were identified as the core evidence base. These key publications were selected because they represent diverse muscle groups, populations, and SWE protocols, collectively illustrating the evolution of the field from early feasibility studies to recent diagnostic applications.

## 3. Biomechanical Principles of Muscle Elastography Versus Conventional Ultrasound

Sarcopenia histological changes involve muscle fat degeneration (fat infiltration), loss of contractile tissue (decrease in muscle mass), and fibrosis (including extracellular matrix changes such as collagen deposition) [14,15]. These processes collectively alter passive and active mechanical properties, with opposing effects on tissue stiffness: fibrosis and increased collagen tend to increase stiffness [16], while myosteatosis and loss of contractile tissue tend to reduce it [17]. Shear-wave elastography therefore captures a composite signal reflecting fibrosis, fat replacement, and residual contractile tissue. Importantly, despite the presence of fibrosis, overall muscle stiffness measured by SWE in sarcopenic individuals is typically reduced compared with healthy controls, suggesting that the combined effects of fat infiltration and muscle atrophy outweigh the stiffening effect of fibrosis on the passive resting state [18]. This reduction in stiffness correlates positively with appendicular skeletal muscle mass index, grip strength, and gait speed, indicating that SWE not only reflects structural integrity but also serves as a functional biomarker [18]. Thus, by quantifying these mechanical properties, SWE provides an objective assessment of one specific facet of muscle quality—the passive biomechanical properties of muscle tissue—rather than the entire multidimensional construct.

As shown in Table 1, conventional ultrasound evaluates muscle morphology (thickness, CSA, echo intensity) and mainly reflects muscle quantity, with moderate functional relevance and limited sensitivity to early change. Shear-wave elastography quantifies muscle stiffness (SWV, elastic modulus), capturing muscle quality related to fat and fibrosis, detecting earlier deterioration, showing stronger associations with strength and performance, and providing more objective, reproducible measurements across different mechanical states. Limitations of conventional ultrasound have driven growing interest in ultrasound elastography as a bedside biomarker of muscle quality and sarcopenia. Ultrasound elastography estimates tissue mechanical properties by measuring deformation in response to stress. Two main approaches have been used in skeletal muscle including strain imaging and shear-wave elastography. Strain imaging (quasi-static elastography) is a qualitative or semi-quantitative assessment of deformation when tissue is compressed manually or by physiological motion; its use in muscle is largely exploratory and operator-dependent. Shear-wave elastography (SWE): acoustic radiation force generates shear waves; their propagation speed is measured and converted into elastic modulus (kPa) or shear-wave velocity (m/s). SWE is quantitative, can be localized to regions of interest, and is the predominant technique in muscle research [19]. To obtain reliable and quantitative measurements of tissue stiffness via shear-wave elastography, several key technical factors must be controlled during acquisition. Defining and reporting the mechanical state of the muscle is crucial, with common states including complete rest, passive stretch, or defined levels of contraction. The transducer should be aligned to orient the ultrasound beam perpendicular to the muscle fibers and held in gentle contact with the skin to avoid pre-compression that could alter tissue mechanics. The system then emits a focused acoustic radiation force impulse (ARFI) into the tissue, generating transverse shear waves that propagate laterally. The ultrasound system tracks the propagation of these shear waves in real time, and shear-wave velocity (SWV) is calculated. As shear waves propagate faster in stiffer tissues, SWV serves as a direct physical proxy for tissue mechanical properties. Assuming linear, elastic, isotropic behavior, SWV can be converted into Young’s modulus (kPa), as illustrated by the following formula: E = 3μ = 3ρvs^2^ = 3ρ(d/t)^2^ [20,21], where E = Young’s modulus (Pa or kPa), μ = shear modulus (Pa), ρ = tissue density (kg/m^3^), typically assumed ≈ 1000 kg/m^3^, νs = shear-wave velocity (m/s), d = distance traveled by shear wave, t = propagation time. However, skeletal muscle is anisotropic and load-dependent, violating these inherent assumptions [20,22]. Therefore, direct reporting of shear-wave velocity (m/s) is preferable to derived modulus in muscle research, as it represents the raw measured parameter without introducing assumption-related errors [22,23]. This recommendation is further addressed in the Section 6. Experimental studies have confirmed that SWE can reliably capture length-dependent changes in passive muscle stiffness as well as activation-dependent changes during submaximal contractions, supporting its use as a quantitative tool for assessing both passive and active muscle mechanics in vivo [24].

Importantly, SWE measurements are largely operator-independent and do not rely on manual compression, distinguishing SWE from strain elastography and enabling more reproducible assessment of muscle mechanical quality. Muscle is anisotropic and exhibits strong load-dependence. Stiffness varies with fiber orientation, passive stretch, and contraction. Standardization of probe orientation, limb position, and contraction state is therefore essential.

**Figure 1:**
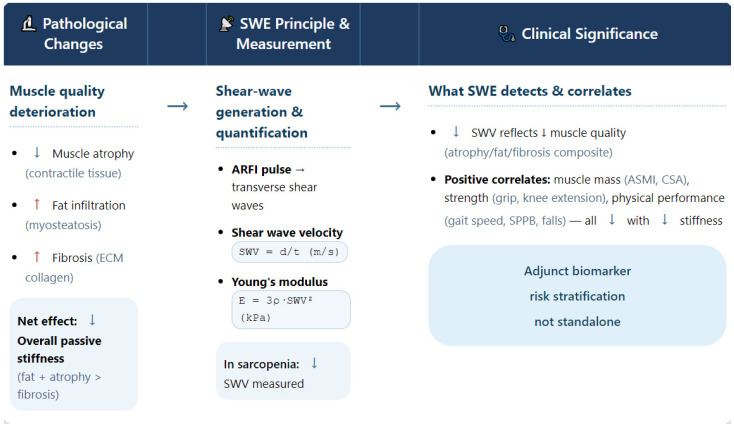
The diagram delineates the three-stage logic underlying SWE application in sarcopenia. Left: Age-related muscle deterioration (atrophy, fat infiltration, fibrosis) leads to a net reduction in passive stiffness, as the softening effects of fat and atrophy predominate over fibrosis. Center: SWE quantifies tissue stiffness via shear wave velocity (SWV), derived from acoustic radiation force impulse (ARFI)—generated shear waves. Right: Lower SWV reflects impaired muscle quality and correlates positively with declines in muscle mass (ASMI, CSA), strength (grip, knee extension), and physical performance (gait speed, SPPB, falls). The bottom box summarizes the current clinical role of SWE as an adjunctive biomarker for risk stratification, not a standalone diagnostic tool.

The integrated framework illustrated in Figure 1 summarizes the biomechanical rationale connecting histopathological alterations to SWE-derived metrics and their established associations with functional outcomes.

Figure 1 Conceptual framework linking skeletal muscle pathology, shear-wave elastography (SWE) measurements, and clinical correlates in sarcopenia.

Building on this conceptual framework, Table 1 provides a detailed comparison between conventional B-mode ultrasound and SWE in terms of their primary measurements, diagnostic value, and clinical utility.

Based on these technical principles, SWE transcends conventional imaging to offer a quantitative biomarker of muscle biomechanical properties. This capability aligns directly with the need to assess muscle quality within contemporary sarcopenia frameworks. Consequently, a growing body of observational research has sought to validate the clinical relevance of SWE by examining its association with sarcopenia-defining parameters and functional outcomes.

## 4. Evidence from Observational Studies in Aging Populations

### 4.1. Age-Related Changes in Muscle Stiffness

Several cross-sectional studies have demonstrated that SWE is sensitive to age-related changes in muscle mechanical properties. Early work by Alfuraih et al. examined lower-limb muscles across different age groups and found that resting stiffness of the quadriceps and gastrocnemius decreased with age [18]. Additionally, lower stiffness was correlated with reduced muscle strength, suggesting that decline in stiffness may reflect underlying loss of contractile tissue. Sendur et al. similarly reported significant inverse correlations between age and gastrocnemius stiffness in both relaxed and contracted states, although the relative increase in stiffness from relaxation to contraction remained preserved across age groups [28]. Consistent with these findings, Do et al. assessed stiffness of the tibialis anterior and medial gastrocnemius at rest and during contraction in young and older adults using both SWE and a myotonometer [29]. They found that older adults exhibited significantly lower stiffness both at rest and during contraction, and the stiffness increase rate was also reduced in the older group. Moreover, Prell et al. reported that shear-wave velocity in major muscle groups—including the quadriceps, hamstrings, and biceps brachii—tends to decline with age, with significant reductions in older participants and consistent associations with frailty measures [30]. However, more recent work emphasizes that age-related changes are not uniform across all muscles or protocols. Han et al. highlighted that SWE metrics of the rectus femoris and gastrocnemius correlated with muscle mass, strength, and gait speed and were reduced in sarcopenic patients with hypertension [31]. Ateş et al. reported that different muscle groups (biceps brachii, rectus femoris, gastrocnemius, hamstrings) show heterogeneous patterns of age-related changes in resting stiffness, with some muscles exhibiting clear reductions in stiffness while others show minimal differences [32]. These findings highlight the importance of muscle-specific reference values, standardized joint positioning, and consistent acquisition protocols. Overall, these studies support that SWE can detect age-related alterations in muscle mechanical properties that are closely linked to strength and performance. Nevertheless, reported findings are substantially influenced by methodological and anatomical factors, including muscle selection, joint positioning, and measurement protocol [33].

### 4.2. SWE in Sarcopenia: Diagnostic and Prognostic Potential

Building on these age-related observations, early narrative reviews proposed elastography as a potential “future biomarker” for sarcopenia, given its ability to quantify muscle stiffness as a surrogate of muscle quality [19]. More recent data have begun to explicitly link elastographic metrics to sarcopenia. For instance, Wang et al. conducted a case-control study (65 sarcopenia patients vs. 65 healthy controls) in which shear wave velocity (SWV) of the tibialis anterior and medial gastrocnemius was significantly reduced in the sarcopenia group and showed strong positive correlations with appendicular skeletal muscle mass index, grip strength, and gait speed [34]. The study further proposed diagnostic cut-off values (3.02 m/s and 2.26 m/s, respectively), with area under the ROC curve exceeding 0.85 for both muscles, suggesting its possible diagnostic utility in this context. However, subsequent studies have reported inconsistent findings, highlighting the need for further validation. Notably, these cutoffs were derived from a single-center case-control design and require external validation before clinical application. According to Okyar Baş et.al., SWE-derived stiffness of the rectus femoris, particularly during passive stretching, was significantly lower in individuals with low handgrip strength and was independently associated with muscle strength, muscle mass, physical performance, and fall history [35]. Therefore, they concluded that SWE is a reliable, noninvasive marker of muscle quality and a promising adjunct for sarcopenia assessment, although further validation of cutoff values is required before routine clinical use [35]. Remuss et al. found that lower rectus femoris stiffness was associated with poor physical performance (SPPB ≤ 7), but no significant difference in stiffness was observed between sarcopenic and non-sarcopenic patients; higher stiffness paradoxically correlated with sarcopenia risk at certain thresholds [36]. Additionally, the sensitivity and specificity at the proposed cut-points, which varied across studies and lacked definitive thresholds, were insufficient for SWE to function as a robust standalone diagnostic tool, while appendicular lean mass measured by DXA remained the preferred measure for sarcopenia [36]. In the same vein, a contemporary review by Galasso et al. concluded that SWE provides complementary information beyond morphometric indices and may enhance risk stratification, but currently lacks validated thresholds, standardized acquisition protocols, and outcome-based cutoffs required for routine clinical diagnosis of sarcopenia [37]. Thus, it is concluded that SWE provides a reliable, noninvasive measure of muscle stiffness and may serve as an effective adjunct tool for sarcopenia assessment. However, diagnostic accuracy of SWE-based measures was limited.

### 4.3. Associations with Functional Performance and Clinical Outcomes

Beyond categorical sarcopenia, several investigations have examined relationships between elastographic measures and continuous functional outcomes. Specifically, SWE-derived stiffness correlates with grip strength, knee extension strength, gait speed, and chair-stand time [18]. Reviews of muscle imaging in sarcopenia show that stiffness adds complementary information, especially in early disease or in “sarcopenic obesity,” where mass may be preserved despite severe quality loss [38]. Further innovating methodology, Tang et al. performed dynamic SWE of the flexor digitorum superficialis during maximal grip contraction in older inpatients [39]. They introduced standardized muscle contractile stiffness (peak SWV/peak grip strength), which showed significant negative correlations with physical performance scores, particularly in men. While innovative, this approach requires validation in larger cohorts and correlation with longitudinal outcomes such as incident disability. Moreover, lower rectus femoris stiffness assessed by SWE was independently associated with reduced muscle strength, physical performance, disease severity, and inflammatory biomarkers, and showed superior predictive performance for COPD patients with sarcopenia compared with conventional ultrasound metrics [40]. Similarly, in geriatric outpatients, decreased gastrocnemius stiffness—particularly during passive stretching—was independently associated with falls, supporting muscle stiffness as an early marker of adverse outcomes distinct from muscle mass and strength [41]. Furthermore, in patients with cardiovascular disease, combining conventional ultrasound with SWE of the rectus femoris and vastus intermedius enabled accurate identification of sarcopenia, with reduced shear-wave velocity reflecting concomitant losses in muscle mass, strength, and gait speed, underscoring the complementary role of SWE in risk stratification and early detection of muscle dysfunction in chronic disease settings [42]. However, few longitudinal studies link baseline elastography to hard outcomes such as falls, fractures, hospitalization, or mortality. Most data are cross-sectional, limiting causal inference.

### 4.4. SWE in High-Risk Clinical Populations

Elastography has also been specifically studied in clinical populations in whom sarcopenia is common. Experience gained from SWE-based assessment of sarcopenia can be readily translated to related clinical contexts in which muscle quality is a key concern. For example, in a cohort of older adults with diabetes, Chen and colleagues combined conventional ultrasound with SWE of lower-limb muscles. SWE showed good test-retest reliability. Patients with diabetes had reduced quadriceps stiffness compared with controls, and lower stiffness was associated with decreased isokinetic strength and impaired functional performance, consistent with diabetes-related sarcopenia or pre-sarcopenia [43]. In addition, a recent study in hemodialysis patients found that decreased quadriceps stiffness on ultrasound elastography was strongly associated with sarcopenia, defined by low muscle mass and strength, and with worse physical performance [44]. Similarly, examining shear-wave elastography of the rectus femoris and vastus intermedius, Yin et al. found that patients with intensive care unit-acquired weakness exhibited significantly increased muscle stiffness and marked reductions in pennation angle [45], whereas muscle thickness and cross-sectional area provided limited discriminatory value. Ruiz-Santana et al. also studied SWE of the quadriceps rectus femoris in ICU patients [46]. Their findings illustrated that increased muscle stiffness paralleled the extent of fibrosis, achieving excellent diagnostic accuracy. When combined with superb microvascular imaging and contrast-enhanced ultrasound, SWE provided complementary information on muscle perfusion and quality, supporting its value as a bedside tool for assessing muscle wasting in long-stay critically ill patients. Furthermore, changes in diaphragm shear modulus measured by ultrasound SWE correlate significantly with alterations in transdiaphragmatic pressure, indicating that SWE can noninvasively reflect diaphragm contractile activity in mechanically ventilated patients [47]. In summary, these findings suggest that elastography may help identify sarcopenic patients in populations with high burden of comorbidity and fluid shifts, where DXA or bioimpedance are challenging [44]. Collectively, these studies indicate a general trend associating lower muscle stiffness with sarcopenia or poor function across diverse populations, although inter-study heterogeneity and methodological variations warrant careful interpretation.

Table 2 summarizes the key muscle sites, elastographic metrics, studied populations, and functional outcomes from the observational studies discussed above, highlighting the application of SWE across different clinical and research contexts. This table provides a selected overview of representative studies to illustrate heterogeneity in research methodologies across the field.

### 4.5. Integrative and Multimodal Approaches

Building upon these foundational correlational observations, recent research has progressed toward evaluating the diagnostic and predictive utility of SWE within integrated assessment frameworks. Notably, studies have begun to combine elastographic parameters with conventional ultrasound and clinical data to construct multivariate models. For instance, Wang et al. developed and validated a diagnostic model for sarcopenia incorporating medial gastrocnemius shear-wave velocity, muscle thickness, age, and body mass index [27]. This logistic regression model demonstrated superior discriminatory power compared to any single parameter, highlighting the additive value of SWE in a composite score. Furthermore, advancing beyond traditional regression, Yi et al. employed machine learning fusion techniques to combine rectus femoris SWE, grayscale ultrasound features, and clinical information [48]. Their work demonstrated that score-level fusion of these multimodal data could achieve higher predictive accuracy for sarcopenia than clinical or imaging features alone. Together, these studies signify an important evolution from observing associations to building practical, data-integrated tools that leverage SWE to enhance objective risk stratification.

In summary, the reviewed literature demonstrates that SWE consistently detects age-related declines in muscle stiffness that correlate with strength and physical performance, although the magnitude of change varies by muscle group and protocol. Reduced stiffness is strongly associated with sarcopenia, and some studies have proposed diagnostic cut-offs with excellent accuracy, though these thresholds lack external validation. Furthermore, lower stiffness predicts adverse outcomes including falls and poor performance in high-risk populations. Notably, integrating SWE with conventional parameters enhances predictive accuracy. However, methodological heterogeneity, lack of standardization, and the predominance of cross-sectional designs currently limit clinical translation.

## 5. Limitations

The potential of ultrasound elastography as a diagnostic tool for sarcopenia is currently constrained by several methodological and interpretative challenges, as summarized in Box 1.

Box 1 **What shear-wave elastography can and cannot provide in sarcopenia.**

**Can provide**

- Objective, quantitative assessment of muscle stiffness
- Early detection of muscle quality decline
- Complementary information to muscle mass and strength

**Cannot yet provide**

- Stand-alone diagnosis of sarcopenia
- Universal cutoffs across devices and muscles
- Clear distinction between fibrosis- and fat-driven stiffness changes
- Outcome-validated thresholds linked to patient-important endpoints (e.g., falls, disa-bility, mortality)

Multiple factors hinder translation of elastography into routine sarcopenia diagnosis. EFSUMB and other expert groups have issued general elastography recommendations, but muscle-specific protocols (muscle selection, position, region of interest, and contraction level) still vary widely across studies [22]. Moreover, muscle is anisotropic, viscoelastic, and load-dependent [22]. Small differences in joint angle or residual contraction substantially alter stiffness measurements. Many studies do not rigorously control or report these variables. In addition, inter-device and inter-vendor variability warrant further consideration. Shear-wave velocities and elastic modulus values are not directly comparable across different ultrasound platforms, complicating establishment of universal reference ranges or cutoffs. Beyond inter-vendor discrepancies, protocols for transducer positioning lack consensus. Even using identical hardware, angular or translational misalignment of the probe relative to the muscle-fiber axis can bias elasticity estimates by altering shear-wave propagation. Critically, handheld application of the transducer introduces uncontrolled, variable pre-compression of subcutaneous tissue and muscle, leading to artifactual overestimation of stiffness. This operator-dependent technical confounder directly compromises inter-operator reproducibility and limits comparability of data across sites and timepoints, particularly in multicenter trials and longitudinal monitoring. Furthermore, a fundamental physical constraint lies in the principle of SWE itself. The technique requires shear waves to propagate over a certain lateral distance, typically several millimeters, which intrinsically limits its spatial resolution and capacity for assessing mechanical properties in highly localized regions. This inherent characteristic may hinder detection of focal, early-stage microstructural changes within muscle tissue [8]. Therefore, research is shifting toward emerging wave modalities like longitudinal shear wave (LSW), which may offer a pathway for more localized assessment [8]. Obesity, edema, and subcutaneous emphysema can degrade signal quality. Lastly, stiffness data may result in uncertain biological interpretation. In particular, reduced stiffness in sarcopenia may reflect loss of contractile tissue, but fibrosis and altered passive tension can increase stiffness. Consequently, the same stiffness value may have different implications depending on muscle, disease, and loading state. Because of these limitations, recent geriatric and imaging editorials have cautioned against over-interpreting elastography and have framed it as a promising but immature biomarker that should complement, not replace, existing diagnostic standards [23].

## 6. Future Directions

Future work should focus on transforming elastography from an exploratory research tool into a standardized, outcome-validated biomarker of muscle quality. Acquisition and reporting must first be harmonized across centers, with explicit control of probe pressure, joint position, muscle selection, contraction state, and clear reporting of shear wave speed, given the marked anisotropy and load-dependence of skeletal muscle demonstrated in experimental and in vivo studies [22,23]. Consistent with Section 3, we endorse reporting shear-wave velocity (m/s) directly rather than converting to Young’s modulus under invalid isotropic assumptions. Future protocols should explicitly account for mechanical loading, recognizing that passive stretch and low-level activation substantially alter SWE measures and may confound resting assessments if not standardized [49]. Technological advances such as angle-resolved SWE and time-harmonic elastography enable quantification of anisotropy, tensile elasticity, and temporal variability, offering sensitivity to microstructural and functional changes that may precede overt muscle atrophy in sarcopenia [50]. In parallel, incorporation of viscoelastic and dispersion-related parameters may improve characterization of aging muscle, where fat infiltration, fibrosis, and reduced thickness can bias simple stiffness estimates. Ultimately, large multicenter, cross-vendor longitudinal studies linking SWE metrics to hard clinical outcomes—such as falls, disability, and mortality—are required to establish normative ranges and validated cutoffs, ideally within multimodal models integrating elastography-derived muscle quality with muscle mass and functional performance.

## 7. Conclusions

Ultrasound elastography, particularly shear-wave elastography (SWE), provides a non-invasive bedside measure of skeletal-muscle stiffness and a valuable indicator of muscle quality. What SWE is worth: Lower muscle stiffness measured by SWE is consistently associated with poor strength, impaired performance, and sarcopenia across diverse populations. It serves as a complementary tool that captures aspects of muscle quality distinct from mass alone, offering additive value for risk stratification and early detection of muscle deterioration, particularly in high-risk populations where conventional assessment is challenging. What SWE is not yet worth: Inconsistent methodology, scarce longitudinal evidence, and the lack of validated thresholds currently limit its diagnostic utility. SWE cannot replace established methods such as DXA for diagnosing low muscle mass, nor can it provide universally applicable cut-off values due to inter-device variability and heterogeneous findings across muscle groups and populations. Its biological interpretation remains complex, as reduced stiffness may reflect fat infiltration, loss of contractile tissue, or a combination of both. Current sarcopenia guidelines (e.g., AWGS 2025, EWGSOP2) do not include SWE in their diagnostic algorithms, as evidence is currently insufficient to establish standardized cutoffs or outcome-based validation. In summary, SWE should be considered an adjunctive research tool that enhances risk stratification and mechanistic insight. Protocol standardization, multicenter validation, and multimodal integration are the essential prerequisites for SWE to inform precision sarcopenia care.

## Figures and Tables

**Table 1:** Conventional ultrasound versus shear-wave elastography for evaluating muscle quantity and quality.

	Conventional Ultrasound	Shear-Wave Elastography
**Primary Measure**	Muscle Morphology (size & structure)	Muscle Biomechanics (tissue stiffness)
**Key Parameters**	Muscle Thickness (MT), Cross-sectional Area (CSA), Echo Intensity (EI)	Shear Wave Velocity (SWV), Elastic Modulus (kPa)
**Diagnostic Value**	**Muscle Quantity**—correlates with lean mass (ASMI)	**Muscle Quality**—correlates with strength & physical performance
**Pathology Insight**	Infers myosteatosis indirectly via increased Echo Intensity [25]	Quantifies tissue stiffness, directly linked to fat/fibrosis content
**Early Detection**	Changes evident after significant muscle atrophy [26]	Changes detectable in early-stage muscle quality decline [27]
**Functional Correlation**	Moderate correlate; a proxy via muscle mass	Strong independent functional biomarker (e.g., balance, fall risk)
**Assessment State**	Typically at rest	Multiple states: rest, passive stretch, active contraction
**Output & Reliability**	Parameters require manual measurement; EI is semi-quantitative with high operator variability	Stiffness values are auto-quantified, providing objective data with good inter-operator reproducibility when probe orientation, transducer pressure, muscle state, and limb position are standardized [22,23]

Abbreviations: MT, muscle thickness; CSA, cross-sectional area; EI, echo intensity; SWV, shear wave velocity; ASMI, appendicular skeletal muscle mass index.

**Table 2:** Representative studies using shear-wave elastography for muscle assessment in aging and sarcopenia.

Muscle Group	Specific Muscle	Elastography Metric	Measurement State	Device/ Vendor	Population	Key Findings
Multiple muscles (e.g., quadriceps, hamstrings, BB)	VL, RF, VM, VI, BF, ST, SM, BB	SWV	Rest	Supersonic Imagine Aixplorer	Healthy adults (20–94 y)	16.5% lower stiffness in elderly; SWV correlated with grip strength, gait speed, chair stands [18]
	Gastrocnemius	Elastic modulus	Rest + Passive stretching	Mindray DC80	Geriatric outpatients	independent predictor of muscle strength; associated with falls [35]
Tibialis anterior, Gastrocnemius		SWV	Rest	Mindray Resona 19S	Sarcopenia vs. healthy controls	SWV reduced in sarcopenia; cut-offs 3.02/2.26 m/s; AUC >0.85 [34]
	Flexor digitorum superficialis	SWV	Dynamic contraction	Supersonic Imagine Aixplorer	Hospitalized older adults	Standardized contractile stiffness correlated with SPPB and TUG [39]
	Rectus femoris	Elastic modulus	Rest	Supersonic Imagine Aixplorer	COPD patients	Lower stiffness associated with reduced muscle strength, physical performance, and inflammatory biomarkers [40]
Quadriceps	RF, VL, VM, VI	SWV	Rest	GE LOGIQ E10	Patients with Type 2 Diabetes	Sarcopenia prevalence, functional decline [43]
Rectus femoris, Vastus intermedius		Elastic modulus	Rest	GE LOGIQ E9	ICU patients (ICUAW)	Increased stiffness in ICUAW; combined with pennation angle improved diagnostic accuracy [45]

Abbreviations: SWV, shear wave velocity; BB, biceps brachii; VL, vastus lateralis; RF, rectus femoris; VM, vastus medialis; VI, vastus intermedius; BF, biceps femoris; ST, semitendinosus; SM, semimembranosus; COPD, chronic obstructive pulmonary disease; ICUAW, intensive care unit acquired weakness; SPPB, short physical performance battery; TUG, timed up-and-go test; AUC, area under the curve.

## Data Availability

No new data were created in this study.
